# Role of Root Exudates in Cadmium Accumulation of a Low-Cadmium-Accumulating Tobacco Line (*Nicotiana tabacum* L.)

**DOI:** 10.3390/toxics11020141

**Published:** 2023-02-01

**Authors:** Huagang Huang, Runze Lu, Juan Zhan, Jinsong He, Yong Wang, Tingxuan Li

**Affiliations:** 1 College of Resources, Sichuan Agricultural University, 211 Huimin Road, Chengdu 611130, China; 2 CAS Key Laboratory of Soil Environment and Pollution Remediation, Institute of Soil Science, Chinese Academy of Sciences, Nanjing 210008, China; 3 Sichuan Provincial Tobacco Company Liangshanzhou Company, 432 Sanchakou East Road, Xichang 615000, China

**Keywords:** cadmium, tobacco, accumulation ability, root exudates, organic acid, amino acid

## Abstract

Root exudates are tightly linked with cadmium (Cd) uptake by the root and thus affect plant Cd accumulation. A hydroponic experiment was carried out to explore the role of root exudates in Cd accumulation of a low-Cd-accumulating tobacco line (RG11) compared with a high-Cd- accumulating tobacco line (Yuyan5). Greater secretion of organic acids and amino acids by the roots was induced by an exogenous Cd addition in the two tobacco lines. The concentration of organic acid secreted by RG11 was only 51.1~61.0% of that secreted by Yuyan5. RG11 roots secreted more oxalic acid and acetic acid and less tartaric acid, formic acid, malic acid, lactic acid, and succinic acid than Yuyan5 under Cd stress. Oxalic acid accounted for 26.8~28.8% of the total organic acids, being the most common component among the detected organic acids, and was significantly negatively correlated with Cd accumulation in RG11. Propionic acid was only detected in the root exudates of RG11 under Cd stress. Lactic acid was positively linked with Cd accumulation in Yuyan5, being less accumulated in RG11. Similarly, RG11 secreted more amino acids than Yuyan5 under Cd stress. Aspartic acid, serine, and cysteine appeared in RG11 when it was exposed to Cd. Lysine was the most secreted amino acid in RG11 under Cd stress. RG11 roots secreted less lysine, histidine, and valine, but more phenylalanine and methionine than Yuyan5 under Cd stress. The results show that organic acids and amino acids in root exudates play a key role in Cd uptake by the root, and this contribution varied with cultivar/genotype. However, further research is still needed to explore the mechanisms underlying low Cd translocation to the leaf, which may be the key contribution of low Cd accumulation in RG11 to the security of tobacco leaf.

## 1. Introduction

Cadmium (Cd) is a widespread heavy metal and is released into the environment as a consequence of industrial and agricultural activities [1,2]. The main effects of Cd toxicity are reflected in plant growth inhibition and the destruction of physiological biochemical processes such as nutrient or water uptake, photosynthesis, oxidation resistance, and cell division [3,4]. Due to its high mobility and bioavailability, Cd is readily taken up by roots and accumulated in plants [5,6]. Therefore, selection and breeding of low-Cd cultivars is a promising method to reduce Cd accumulation in plants [7]. It has been reported that the accumulation of Cd in plants is closely related to its uptake characteristics [8]. Apart from the influence of the root system, root exudates play a key role in deeply solubilizing metals in the rhizosphere and affecting Cd availability in soils and Cd uptake by the roots [1,9]. 

Numerous studies have demonstrated that organic acids and amino acids play an important role in tolerating and accumulating heavy metals [10,11]. However, the role of the complexation of organic acids and amino acids secreted by roots in Cd uptake and accumulation in plants remains debated. Exogenous acetic acid and malic acid can significantly reduce the toxicity of Cd in soils and increase Cd uptake by sunflower roots [12]. Proline and histidine secreted by the roots enhance the accumulation of Cd in Cd-enriched black nightshade (*Solanum nigrum* L.) [13]. On the other hand, low-Cd-accumulating potatoes accumulate less Cd than high-Cd cultivars, but their root exudates more organic acids than high-Cd potatoes [14]. The root exudates of different plants or different accumulation types of the same plant play different roles in Cd uptake and accumulation.

Tobacco (*Nicotiana tabacum* L.), an important economic crop, can greatly bioconcentrate Cd and translocate Cd into leaves, producing the material used for cigarettes [15,16]. In a previous study, RG11, a flue-cured tobacco, was identified as a low-Cd-accumulating tobacco line fit for safe production from Cd-contaminated soils [17]. RG11 exhibited lower Cd uptake and leaf Cd accumulation compared with the high-Cd tobacco line Yuyan5 [18]. To date, the exudation properties of tobacco cultivars in response to Cd stress are still unclear. One hypothesis is that RG11 secrets lower amounts of organic and amino acids and has a weaker solubilization effect on Cd. Therefore, the objective of this study was to investigate the mechanism underlying differences in Cd uptake by comparing the different responses of organic acids and amino acids in the root exudates of a low-Cd-accumulating tobacco line (RG11) and high-Cd-accumulating tobacco line (Yuyan5).

## 2. Materials and Methods

### 2.1. Plant Material

In the present study, two different Cd-accumulating tobacco lines with similar growth periods were used. In a previous study, RG11 was identified as a low-Cd-accumulating tobacco line and Yuyan5 as a high-Cd-accumulating tobacco line [19]. The tobacco seeds were soaked in 30% H_2_O_2_ for 30 min and 0.1% NaClO_3_ for 1 h for surface sterilization. After being washed thoroughly with deionized water, the seeds were sowed in a culture dish (length 50 cm × width 30 cm × height 7 cm) at 30 °C and 60% relative humidity. After germination, the seeds were transferred to a seed tray filled with culture medium of peat, perlite and vermiculite (5:1:1, *v*/*v*/*v*). Finally, when the fifth leaf unfolded, healthy and uniform seedlings from the two tobacco lines were chosen for the hydroponics experiment.

### 2.2. Experimental Design

Cd treatments were designed as 0, 0.5 and 1.0 mg Cd L^−1^ in a hydroponic experiment and supplied with cadmium chloride hydrate (CdCl_2_·2.5H_2_O). 

Completely random permutation was used with three replicates per treatment. Healthy and uniform tobacco line seedlings with at least five leaves were transferred to hydroponic containers (8 L, length 40 cm × width 20 cm × height 10 cm), and covered with plastic plates with one smooth, round hole (one plant per hole) in a green house. The day/night temperature was 30/20 °C, and the humidity was approximately 80%. Prior to Cd treatment, the seedlings were pre-cultured in basal nutrient solution for two weeks. The nutrient media were renewed every five days. The composition of the nutrient solution was as follows: 4.00 mmol L^−1^ calcium nitrate tetrahydrate (Ca(NO_3_)_2_·4H_2_O), 5.00 mmol L^−1^ potassium nitrate (KNO_3_), 1.00 mmol L^−1^ ammonium nitrate (NH_4_NO_3_), 1.00 mmol L^−1^ monobasic potassium phosphate (KH_2_PO_4_), 4.10 mmol L^−1^ magnesium sulfate (MgSO_4_), 20.00 mmol L^−1^ iron sulfate heptahydrate (FeSO_4_·7H_2_O), 22.19 mmol L^−1^ ethylenediamine tetraacetic acid disodium salt (EDTA-Na_2_), 5.00 μmol L^−1^ potassium iodide (KI), 0.10 mmol L^−1^ boric acid (H_3_BO_3_), 0.15 mmol L^−1^ manganese sulfate (MnSO_4_), 0.05 mmol L^−1^ zinc sulfate (ZnSO_4_), 1.03 μmol L^−1^ sodium molybdate (Na_2_MoO_4_), 0.15 μmol L^−1^ copper sulfate (CuSO_4_), 0.19 μmol L^−1^ cobalt chloride (CoCl_2_). The pH was adjusted to 6.5 using 0.10 mol L^−1^ hydrochloric acid (HCl) or sodium hydroxide (NaOH). 

### 2.3. Cd Treatment and Root Exudate Collection

Before the collection of root exudates, the tobacco lines were exposed to a nutrient solution containing different concentrations of Cd for 6 days, with 6 plants in per container. Then, three seedlings from each treatment were gently removed from the hydroponic containers. Root exudates were collected using the method described by Tao et al. [20] with minor modifications. After rinsing them thoroughly with tap water and deionized water, the roots were placed in 0.2 mmol L^−1^ calcium chloride (CaCl_2_) solutions for 2 h and 30 mg L^−1^ chloramphenicol solution for 30 min to inhibit bacterial growth. Subsequently, each plant was transferred to a dark brown glass bottle containing 250 mL of deionized water for 24 h in the growth chamber. Then, they were diluted with deionized water to 250 mL and eluted by a pre-activated SAX solid-phase extraction column. The eluent was freeze-dried (FreeZone 12, Labconco Corp., Kansas City, MO, USA) and filtered through 0.2 μm membranes for analysis. 

### 2.4. Root Exudate Analysis

The organic acids were detected by high-performance liquid chromatography (HPLC) (Agilent 1260, Santa Clara, CA, USA), with an ion-exchange analytical column C18 (5 μm, 4.6 mm × 250 mm). The mobile phase was KH_2_PO_4_ and methanol (97:3, *v*/*v*; pH 2.5), and the flow rate was set at 0.8 mL min^−1^. Organic acids were detected using a UV detector (Shimadzu SPD-10ATvp) at 210 nm. Root exudates collected from the solution without Cd exposure were regarded as a control and were standardized regarding treatment time and root fresh weight (mg 24 h^−1^ g^−1^ FW), which was measured immediately after the treatment. Standard organic acids including oxalic acid, tartaric acid, formic acid, malic acid, lactic acid, acetic acid, maleic acid, propionic acid, succinic acid, citric acid, malonic acid, and butanoic acid were subjected to the same conditions as the controls. Identification of the organic acids was performed by comparing their peak areas and retention times with those of established standards. 

The amino acids were detected using an L-8900 amino acid analyzer (Hitachi, Ibaraki, Japan). The standard chromatographic column (ID 4.6 mm × 60 mm) was selected, with a working temperature of 50 °C and detection wavelength of 510 nm. The mobile phase was trisodium citrate dehydrate (pH 3.2–4.9) and the flow rate was 0.4 mL min^−1^. The reaction temperature was 135 °C and the reaction solution was a ninhydrin coloring solution kit. Standard amino acids including aspartic acid (Asp), tryptophan (Trp), threonine (Thr), serine (Ser), glutamic acid (Glu), glycine (Gly), alanine (Ala), cysteine (Cys), valine (Val), methionine (Met), isoleucine (Ile), leucine (Leu), tyrosine (Tyr), phenylalanine (Phe), lysine (Lys), histidine (His), and arginine (Arg) were set as the controls. The amino acid contents of the collected solutions were directly determined via data analysis with the L-8900 AAA System Manager.

### 2.5. Biomass and Cd Concentrations of Tobacco Lines

Following the collection of root exudates, samples were divided into shoots and roots. The roots were soaked in 20 mmol L^−1^ Na_2_-EDTA for 30 min to remove surface-adsorbed Cd and rinsed thoroughly with deionized water. Then, all samples were de-enzymed at 105 °C for 30 min and dried at 75 °C to a constant weight. The dried samples were weighed and smashed with a stainless steel grinder (FW-100, China). Then, 0.15 g of ground samples was digested with nitric acid (HNO_3_) and perchloric acid (HClO_4_) (5:1, *v*/*v*) for 8 h and diluted in a 25 mL volumetric flask with deionized water. Cd concentrations in the digested solutions were determined using an AA900T flame atomic absorption spectrophotometer (PerkinElmer, Waltham, MA, USA). In the determination process, an internal reference was used. The limit of quantification for Cd is 0.001 μg mL^−1^. 

### 2.6. Statistical Analysis

Data were analyzed statistically using the two-way ANOVA. Differences among Cd treatments or tobacco lines were tested using the least significant difference (LSD) test for a *p* value < 0.05. All data are expressed as means with standard errors (SEs). Statistical analysis and graphical work were performed on DPS 11.0, Origin 9.0, and Excel 2013. SPSS software 22.0 was used for stepwise regression analysis and path analysis.

## 3. Results

### 3.1. Plant Biomass

**Figure 1:**
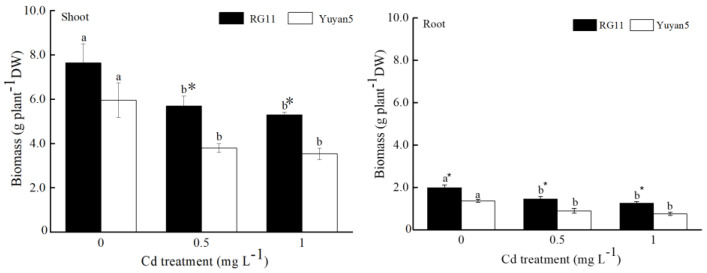
Biomass in shoot and root of two tobacco lines grown in different Cd treatments. The data are the means of three independent replicates. Error bars indicate standard deviation. Different letters above the bars indicate significant level at *p* < 0.05 among Cd treatments within same tobacco line. * indicates significant level at *p* < 0.05 among the tobacco lines within same Cd treatment.

The biomass of the two tobacco lines significantly decreased after treatment with Cd (Figure 1). The shoot and root biomasses of RG11 under Cd stress were 25.5~40.6% lower than those of the control, while the shoot and root biomasses of Yuyan5 were 27.0~44.7% lower than those of the control. Under 0.5 and 1 mg L^−1^ Cd treatments, the shoot and root biomasses of RG11 were 1.50~1.51 and 1.62~1.66 times higher than those of Yuyan5, respectively. These values indicate that the low-Cd-accumulating tobacco line has higher Cd tolerance than the high-Cd-accumulating tobacco line.

### 3.2. Cd Concentration

**Figure 2:**
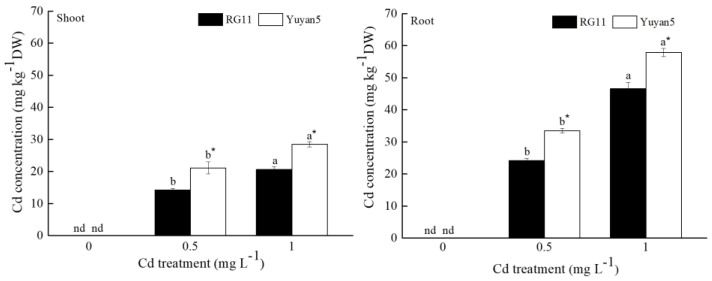
Cd concentrations in shoot and root of two tobacco lines grown in different Cd treatments. The data are the means of three independent replicates. Error bars indicate standard deviation. Different letters above the bars indicate significant level at *p* < 0.05 among Cd treatments within same tobacco line. * indicates significant level at *p* < 0.05 among the tobacco lines within same Cd treatment. nd means not detected.

A significant increase in Cd concentrations was observed in the shoot and root of the two tobacco lines with larger exogenous cadmium doses in the nutrient solution, reaching the highest values under the highest Cd dose (Figure 2). The Cd concentration in the shoot of the two tobacco lines was lower than that in the root. In comparison with Yuyan5, there were significantly lower Cd concentrations in the shoot and root of RG11. Cd concentrations in the shoot and root of RG11 were 67.3~72.4% and 72.2~80.5% lower than those in Yuyan5, respectively.

### 3.3. Organic Acid in Root Exudates

Under Cd treatments, the total organic acids secreted by the two tobacco lines increased dramatically compared with those secreted by the control (Table 1). RG11 secreted a 51.1~61.0% lower concentration of total organic acids than Yuyan5 under the same Cd treatment.

There were more kinds of organic acids in the root exudates of the two tobacco lines under Cd treatments than in the control. Tartaric acid and malic acid were secreted by both tobacco lines under Cd stress, while propionic acid was only detected in the root exudates of RG11. Unlike in Yuyan5, propionic acid may be involved in Cd uptake and accumulation in RG11. The organic acid in the root exudates of RG11 after the Cd treatments had similar compositions, with oxalic acid accounting for the most common acid at approximately 26.8~28.8% of the total organic acid. Apart from maleic acid, the concentrations of tartaric acid, formic acid, malic acid, lactic acid, and succinic acid in RG11 were 15.2~75.5% lower than those in Yuyan5. At 1 mg L^−1^ Cd, the concentrations of oxalic acid and acetic acid in the root exudates of RG11 were significantly higher than in those of Yuyan5, being 1.63 and 2.14 times higher, respectively.

Based on the results of the stepwise regression analysis and path analysis (Table 2 and Table 3), a significant positive correlation between propionic acid and succinic acid concentrations and Cd accumulation in plants was observed in RG11 under Cd treatments. On the contrary, the concentration of oxalic acid in RG11 was significantly negatively correlated with Cd accumulation in plants.

### 3.4. Amino Acid in Root Exudates

Cd stress significantly promoted the secretion of total amino acids in the roots of the two tobacco lines (Table 4). With increasing Cd doses, the total amino acid concentrations in the root exudates of RG11 increased significantly in comparison with those of the control, being 1.49 and 6.53 times higher. Meanwhile, the concentrations of total amino acids were 1.53 and 6.02 times higher than those of the control. The secretion of total amino acids by RG11 was 71.2~72.8% that of Yuyan5 under the same Cd stress. Asp was secreted by both tobacco lines under Cd stress, while Ser and Cys were only detected in the root exudates of RG11 with increasing Cd doses. The Asp, Gly, Met, and Phe concentrations of RG11 were 1.34~11.50 times higher than those of Yuyan5 under all Cd treatments. Under Cd stress, RG11 secreted significantly lower amino acid amounts than Yuyan5, in addition to Ala, Tyr, and the other four abovementioned amino acids. 

Lys, the main amino acids secreted by RG11, accounted for approximately 13.0~19.2% of the total amino acids. The Lys concentrations in the root exudates of RG11 were positively correlated with Cd accumulation in plants under Cd stress (Table 5 and Table 6). Additionally, the Phe concentrations of RG11 were significantly higher than those of Yuyan5 and also remarkably positively correlated with Cd accumulation in RG11. As for Yuyan5, Val and Tyr had positive correlations with Cd accumulation in plants.

## 4. Discussion

### 4.1. Response of Plants to Cd

The composition and concentration of organic acids secreted by plant roots will change under exposure to Cd stress, reflecting plants’ response to Cd [21,22]. Organic acids secreted by the roots are closely related to Cd detoxification, absorption, and accumulation in plants [14,23]. The addition of Cd promotes the secretion of organic acids from the roots of maize, sunflower, and awn grass and reduces Cd’s toxicity to plants. Furthermore, the secretion of organic acids increased significantly with increasing Cd concentrations [12,24,25]. Organic acids secreted from the roots can activate Cd and affect the bioavailability in soil plant systems [26]. In our study, Cd stress induced the secretion of organic acids from the roots of two tobacco lines, but there was a significant difference between them (Table 1). The organic acid concentrations seem to depend on plants’ level of Cd tolerance [27]. Compared with the high-Cd-accumulating tobacco line, the root system of the low-Cd-accumulating tobacco line secreted significantly smaller amounts of organic acids, thus limiting its Cd uptake and accumulation. Similar results were found for low-Cd-accumulating amaranth (*amaranth manfostanus* L.), low-Cd-accumulating hot pepper (*Capsicum annuum* L.), and the non-hyperaccumulating ecotype of *Sedum alfredi* Hance (NHE) [28,29,30]. These plants’ low organic acid secretion in the roots is closely related to lower Cd accumulation. It can be concluded that the root system of the low-Cd-accumulating tobacco line secreted a smaller amount of organic acids in the root exudates under all Cd treatments, and hence it demonstrated low-Cd-uptake characteristics. 

Oxalic acid, the most common dicarboxylic acid, has a strong ability to bind with heavy metal ions. Alleviating the oxidative damage caused by abiotic stress can affect heavy metal accumulation in plants. However, significant variation in Cd accumulation in different plants was observed due to the secretion of oxalic acid [31]. The hyperaccumulating ecotype of *S. alfredii* secreted more oxalic acid compared with NHE, resulting in a promotion of Cd uptake and accumulation in the hyperaccumulating ecotype [20]. However, the opposite phenomenon was observed in a Cd-tolerant tomato, whose roots secreted more oxalic acid accompanied by lower Cd accumulation [32]. This may be attributed to the fact that the role of organic acids varies with plant species. In this study, oxalic acid accounted for the largest proportion of organic acids in the low-Cd-accumulating tobacco line, which had higher concentrations of this acid than the high-Cd-accumulating tobacco line (Table 1). 

The results of stepwise regression analysis and path analysis show there was a negative relationship between oxalic acid and Cd accumulation in RG11. Oxalic acid can also bind with divalent Cd in the rhizosphere to prevent roots from absorbing Cd from soils [32]. The high oxalic acid secretion of RG11 may contribute greatly to lower Cd accumulation in the roots as well as a higher Cd tolerance. Further research is necessary to explore the mechanisms underlying low Cd translocation to the leaf, which is key for the low Cd accumulation in RG11. As a monocarboxylic acid, propionic acid showed a lower ability to bind with Cd due to having fewer binding sites than dicarboxylic organic acids [33]. Compared with monocarboxylic acid, succinic acid (dicarboxylic acid) secreted by maize has more negative charges, providing more binding sites and enhancing Cd binding affinity [27]. The results show that succinic acid plays a direct positive role in Cd accumulation in the low-Cd-accumulating tobacco line. The amounts of succinic acid secreted by the root system of the low-Cd-accumulating tobacco line were significantly lower than those of the high-Cd-accumulating tobacco line (Table 1 and Table 3). With the increase in Cd concentrations, the succinic acid secreted by the root system of RG11 could promote Cd accumulation. The Cd accumulation ability of the low-Cd-accumulating tobacco line was weaker than that of the high-Cd-accumulating tobacco line. Lactic acid was the only organic acid that plays a direct role in Cd accumulation in the high-Cd-accumulating tobacco line (Table 3). Under Cd stress, the roots of the low-Cd-accumulating tobacco line secreted a lower amount of lactic acid, leading to its low Cd accumulation. Further experiments are necessary to clarify the specific mechanism of the organic acids acting in Cd uptake and accumulation in the low-Cd-accumulating tobacco line.

### 4.2. The Effect of Amino Acids on Cd Accumulation 

As a product of plant metabolism, amino acids play an important role in mineral nutrient uptake, heavy metal detoxification, and cell osmotic regulation [34,35]. In our research, there were significant differences in the composition and quantities of amino acids secreted by roots between the two tobacco lines (Table 4). With the increase in Cd concentrations, the total amount of amino acids secreted by the roots of the two tobacco lines increased significantly. This is consistent with previous research on soybean that showed that Cd stress accelerates the secretion of amino acids by the root [36]. The total amounts of amino acids secreted by the roots of low-Cd-accumulating tobacco line were significantly lower than those secreted by the high-Cd-accumulating tobacco line (Table 4). The variation in amino acid concentrations in the root exudates of the two tobacco lines may be due to differences in Cd uptake and accumulation. Amino acids can chelate with Cd ions, thus reducing the migration of Cd to plants and its toxic actions [37]. Lys and His secreted by plant roots play a key role in chelating, activating, and absorbing heavy metals [38,39]. Lys was the main amino acid secreted by the roots of the low-Cd-accumulating tobacco line. The secretion of Lys by the roots of the low-Cd-accumulating tobacco line was significantly lower than that of the high-Cd-accumulating tobacco line (Table 4). According to path analysis, Lys had the greatest direct effect on Cd accumulation in the low- Cd-accumulating tobacco line (Table 6). The Cd accumulation of the low-Cd-accumulating tobacco line was lower and related to lower Lys secretion by the roots.

His accounted for the largest proportion of amino acids secreted by the high-Cd-accumulating tobacco line. Under Cd stress, the concentration of His secreted by the roots of the low-Cd-accumulating tobacco line was significantly lower than that of the high- Cd-accumulating tobacco line. His secretion was also one of the factors that affected Cd uptake by the roots between the two tobacco lines. Khodamoradi et al. found that His significantly increased Cd accumulation in rye (*Triticosecale* cv. Elinor) and durum wheat (*Triticum aestivum* L. cv. Back Cross Rushan) by adding His to an exogenous medium [40]. Phe is another major factor that contributed to Cd accumulation in the low-Cd-accumulating tobacco line. Under Cd treatment, the Phe secretion of the root system of RG11 was higher than that of Yuyan5. Isoflavones such as plant defensins and lignin synthesized by the phenylalanine metabolic pathway can effectively remove peroxides produced under stress and improve plant tolerance [41]. Met metabolites also contribute to scavenging free radicals, resisting membrane lipid peroxidation, and enhancing plant tolerance [42]. In our study, the concentration of methionine secreted by the roots of the low-Cd-accumulating tobacco line was significantly higher than that of the high-Cd-accumulating tobacco line. The differences in the secretion of Phe and Met significantly contributed to the difference in Cd tolerance between the two tobacco lines. In addition, Val played a direct and positive role in Cd accumulation in the high-Cd-accumulating tobacco line (Table 6). Compared with the high-Cd-accumulating tobacco line, the roots of the low-Cd-accumulating tobacco line secreted a lower amount of Val by roots, thus limiting Cd accumulation in plants.

## 5. Conclusions

The low-Cd-accumulating flue-cured tobacco line RG11 showed less secretion of organic acids and amino acids than Yuyan5 under Cd stress. In particular, RG11 roots secreted less tartaric acid, formic acid, malic acid, lactic acid, succinic acid, lysine, histidine, and valine than Yuyan5. The low secretion of organic acids and amino acids of RG11 contributes greatly to low Cd uptake by the roots. Oxalic acid was the most frequently detected organic acid in the root exudates of RG11, having significant positive correlation with Cd accumulation. Oxalic acid plays an important role in promoting Cd accumulation in the roots and the Cd tolerance of RG11. The contribution of amino acids varies with the tobacco line. This study investigated tobacco lines grown in a mineral solution rather than in soil for only 6 days due to the difficulty of establishing a complete collection of root exudates in soils. Further research is necessary to explore the mechanisms of low Cd accumulation in a natural environment and lower Cd translocation to the leaf, which may play significant roles in low Cd accumulation in RG11 for food safety.

## Figures and Tables

**Table 1:** The composition and concentrations of organic acids in root exudates of two tobacco lines grown in different Cd treatments.

Cd Treatment (mg L^−1^)	Line	Organic Acids Concentration (mg 24 h^−1^ g^−1^ FW)									
		Oxalic Acid	Tartaric Acid	Formic Acid	Malic Acid	Lactic Acid	Acetic Acid	Maleic Acid	Propionic Acid	Succinic Acid	Total
0	RG11	8.82 b *	nd	0.15 b	nd	0.97 c	0.67 b	0.04 b	0.25 b	2.30 c	13.20 c
0.5		10.84 b	5.07 b	0.72 b	1.59 b	9.01 b	0.83 b	0.15 a	0.43 b	8.42 b	37.06 b
1		20.69 a *	9.75 a	2.03 a	7.29 a	16.88 a	2.71 a *	0.39 a	1.35 a	14.05 a	75.14 a
0	Yuyan5	4.79 b	nd	0.66 b *	nd	nd	0.43 b	0.16 b *	nd	18.62 c *	24.66 c *
0.5		9.68 a	7.48 b *	4.60 a *	7.70 b *	16.66 b *	0.98 ab	0.26 b	nd	26.28 b *	73.64 b *
1		12.69 a	14.81 a *	5.73 a *	13.17 a *	22.41 a *	1.38 a	0.57 a	nd	55.79 a *	126.55 a *

Note: The data are the average of three replicates. nd means not detected. The lowercase letters refer to significant differences among the Cd treatments within same tobacco line. The * indicates significant level at *p* < 0.05 among the tobacco lines within same Cd treatment.

**Table 2:** Stepwise regression analysis between concentrations of organic acids in root exudates and Cd accumulation of two tobacco lines under different Cd treatments.

Line	Model	Parameter	Equation	*r* ^2^	F	n
RG11	1	Propionic acid	*y* = −19.17 + 13.38*x*_1_	0.906	0.000	9
	2	Propionic acid	*y* = 17.19 + 20.40*x*_1_ − 7.01*x*_2_	0.954	0.029	9
		Oxalic acid				
	3	Propionic acid	*y* = 45.15 + 20.16*x*_1_ − 11.86*x*_2_ + 57.95*x*_3_	0.985	0.014	9
		Oxalic acid				
		Succinic acid				
Yuyan5	1	Lactic acid	*y* = −0.83 + 7.36*x*_1_	0.975	0.000	9

**Table 3:** Path analysis between concentrations of organic acids in root exudates and Cd accumulation of two tobacco lines under different Cd treatment.

Line	Independent Variable	*r*	Path Coefficient	Contribution	Indirect Path Coefficient			
					*x*_1_	*x*_2_	*x*_3_	Total
RG11	*x*_1_ (Propionic acid)	0.958	1.443	1.383	-	−0.849	0.364	−0.485
	*x*_2_ (Oxalic acid)	0.792	−0.926	−0.734	1.323	-	0.395	1.718
	*x*_3_ (Succinic acid)	0.803	0.426	0.342	1.236	−0.859	-	0.377
Yuyan5	*x*_1_ (Lactic acid)	0.989	0.989	0.978	-	-	-	-

**Table 4:** The composition and concentrations of amino acids in root exudates of two tobacco lines grown in different Cd treatments.

Cd Treatment (mg L^−1^)	Line	Amino Acids Concentration (mg 24 h^−1^ g^−1^ FW)																
		Asp	Thr	Ser	Glu	Gly	Ala	Cys	Val	Met	Ile	Leu	Tyr	Phe	Lys	His	Pro	Total
0	RG11	nd	0.03 b	Nd	0.04 c	0.23 c *	0.05 c	nd	0.20 c	0.08 c	0.10 c	0.07 c	0.24 c	0.16 c	0.23 c	0.15 c	0.05 b	1.61 c
0.5		0.02 b	0.02 b	0.01 b	0.69 b	0.74 b *	0.27 b	0.07 b	0.67 b	0.14 b *	1.17 b	1.24 b	0.77 b	1.52 b	1.41 b	1.03 b	0.17 a	10.51 b
1		0.20 a *	0.06 a	0.05 a	1.35 a	1.10 a *	0.35 a	0.18 a	0.99 a	0.48 a *	1.41 a	1.74 a	1.11 a	1.68 a *	2.37 a	1.81 a	0.18 a	15.71 a
0	Yuyan5	nd	0.02 c	0.04 c	0.04 c	0.02 b	0.05 c	0.01 b	0.16 c	nd	0.12 b	0.39 c *	0.25 c	0.15 b	0.21 c	0.86 c *	0.06 b	2.40 c *
0.5		nd	0.04 b *	0.08 a *	0.54 b	0.18 a	0.34 b	0.10 b	1.38 b *	0.08 b	1.58 a *	2.30 b *	0.70 b	1.38 a	1.97 b *	3.95 b *	0.39 a *	14.44 b *
1		0.04	0.09 a	0.06 b	3.74 a *	0.19 a	0.42 a	1.44 a *	1.80 a *	0.15 a	1.58 a	2.76 a *	1.12 a	1.25 a	3.02 a *	4.78 a *	0.39 a *	22.08 a *

Note: The data are the average of three replicates. nd means not detected. The lowercase letters refer to significant differences among the Cd treatments within same tobacco line. The * indicates significant level at *p* < 0.05 among the tobacco lines within same Cd treatment.

**Table 5:** Stepwise regression analysis between concentrations of amino acids in root exudates and Cd accumulation of two tobacco lines under different Cd treatments.

Line	Model	Parameter	Equation	*r* ^2^	F	n
RG11	1	Lys	*y* = −8.83 + 57.64*x*_1_	0.977	0.000	9
	2	Lys	*y* = −13.76 + 38.44*x*_1_ + 34.13*x*_2_	0.987	0.047	9
		Phe				
Yuyan5	1	Val	*y* = −17.86 + 101.33*x*_1_	0.991	0.000	9
	2	Val	*y* = −24.00 + 81.02*x*_1_ + 41.58*x*_2_	0.997	0.012	9
		Tyr				

**Table 6:** Path analysis between concentrations of amino acids in root exudates and Cd accumulation of two tobacco lines under different Cd treatments.

Line	Independent Variable	*r*	Path Coefficient	Contribution	Indirect Path Coefficient		
					*x*_1_	*x*_2_	Total
RG11	*x*_1_ (Lys)	0.990	0.660	0.653	-	0.330	0.330
	*x*_2_ (Phe)	0.976	0.345	0.337	0.631	-	0.631
Yuyan5	*x*_1_ (Val)	0.996	0.796	0.793	-	0.200	0.200
	*x*_2_ (Tyr)	0.962	0.212	0.204	0.750	-	0.750

## Data Availability

The data presented in this study are available within the article.
